# Notes for the study on health systems: multifaceted analysis and tracer indicators

**DOI:** 10.11606/s1518-8787.2020054002055

**Published:** 2020-10-28

**Authors:** Rosana Teresa Onocko-Campos, Gastão Wagner de Sousa Campos, Carlos Eduardo Menezes Amaral, Oswaldo Yoshimi Tanaka

**Affiliations:** I Universidade Estadual de Campinas Faculdade de Ciências Médicas Departamento de Saúde Coletiva CampinasSP Brasil Universidade Estadual de Campinas . Faculdade de Ciências Médicas . Departamento de Saúde Coletiva . Campinas , SP , Brasil; II Universidade Federal do Vale do São Francisco Colegiado de Medicina de Paulo Afonso Paulo AfonsoBA Brasil Universidade Federal do Vale do São Francisco . Colegiado de Medicina de Paulo Afonso . Paulo Afonso , BA , Brasil; III Universidade de São Paulo Faculdade de Saúde Pública São PauloSP Brasil Universidade de São Paulo . Faculdade de Saúde Pública . São Paulo , SP , Brasil

**Keywords:** Primary Health Care, Secondary Health Care, Triangulation of Methods, Outcome and Process Evaluation, Health Care

## Abstract

**OBJECTIVE:**

To present the methodological approach used in a research that analyzed the use and performance of specialized health care, from primary care access, in four major Brazilian cities: Fortaleza (CE), Campinas (SP), São Paulo (SP) and Porto Alegre (RS).

**METHODS:**

Presentation and discussion of the quantitative-qualitative components of the proposed research strategy.

**RESULTS:**

Four tracing conditions were studied: systemic arterial hypertension, high-risk pregnancy, breast cancer and severe mental disorder. For each health condition, indicators were constructed based on health information systems data, pointing out frequencies, temporal trends and local differences. This initial contextualization was enriched with a descriptive-qualitative study of the performance of each municipal health service network. Next, a cross-sectional study was conducted through a survey of 7,053 users of specialized services for each health condition. Finally, in-depth interviews were conducted with key actors to complement selected operational aspects of each municipality’s network. The results of all these data sources were triangulated, allowing us to explore the variability of SUS implementations in different regional scenarios.

**CONCLUSIONS:**

The multifaceted analytical model presented allows us to understand relevant aspects of the Unified Health System performance, paying attention to the singularities, heterogeneities and inequalities that characterize its implementation in Brazil and emphasizing the performance of local networks for the addressed health conditions.

## INTRODUCTION

In our article, we present a combination of mixed and multifaceted methods developed by a multicenter group of Brazilian researchers to analyze the performance and use of specialized care, from primary healthcare (PHC) access, for four tracing conditions in four major Brazilian cities: Fortaleza (CE), Campinas (SP), São Paulo (SP) and Porto Alegre (RS).

According to the World Health Organization, the health systems analysis field remains relatively little explored, particularly in low- and middle-income countries ^1^ . In Latin America, Brazilian scientific production in the health evaluation field stands out, with special interest in the service integration that occurred after the implementation of health care networks (HCN) policies by the government, in the last decade. However, both in Brazilian and international literature, studies on health policies and services field tend to focus on specific programs and actions instead of national or local health systems ^2^ .

When they occur, health systems analyses have been based on different approaches. A recent review pointed out excessive confidence in descriptive methods and cross-sectional studies is an important problem in systems and services studies ^3^ . Among many aspects to be overcome in favor of the accuracy and sophistication of the field, this review points out that most studies include a single type of informant or information level and use data from a single temporal point. Regarding the analysis frameworks, many studies use only descriptive statistics, and the vast majority do not adequately declare their level of sensitivity or statistical power.

Other authors have also drawn attention to the need for researchers to develop new forms of analysis, in order to establish appropriate parameters to represent and explain highly complex scenarios ^4^ . The effects health systems produce cannot be explained by a simple linear causality relation, demanding references that adopt the notion of complex causality, understood as multiple causes that interact and generate (sometimes unpredictable) effects ^5^ .

The requirement to adopt complex causality also derives from the knowledge that actions and policies often affect differently distinct places and times ^5^ . Therefore, health systems studies are required to understand the history and local context of subsystems, since:

[...] when the analysis moves from medical care to more complex levels of health practices organization in municipalities or health districts [...], obscuring the context and historicity of the object, as operated by the supposed paradigm universality, compromises the understanding of the meaning of events ^6^ .

The possibility of generalizing the findings is another challenge to health system research. Drawing conclusions of direct application in the generic set of health systems and subsystems is challenging, especially when considering the heterogeneity of national health systems and their local subsystems and the complex causality that organizes their effects.

Therefore, studies should seek analytical generalization. This type of generalization allows the development of general conclusions that, although derived from singular experiences, provide theoretical insights to be tested in other contexts, guiding both future research and decision-making processes ^7 , 8^

Based on this characterization, our study seeks to present a multifaceted analytical model, developed to investigate the performance of specialized care and its access via PHC in four major Brazilian cities based on tracing diseases.

## RESEARCH STRATEGY

We seek with this article to present a research strategy consistent with the challenges of the field, sharing the experience of researchers from several universities, policy-makers and health professionals from different states of Brazil.

We present our experience as a research strategy, understanding it not only as a study design or a particular method, but as a way to approach the health system combining methods of data collection and sampling in the most appropriate way possible to the purposes and object of our investigation ^5^ . Therefore, we point out some theoretical and methodological premises of the research strategy regarding its purposes and object.

In general, research on health systems and policies have as purpose some quality assessment. However, this intention implies some complicating factors, such as the theoretical plurality of evaluative references, whose distinction begins in the ontological and epistemological perspective adopted, resulting in important methodological implications.

In the field of health evaluation, currents in two distinct ontological and epistemological polarities stand out: the positivist perspective and the interactionist/constructivist approaches. In our research strategy, we recognize the possibility of reducing the distance between the tools of the positivist method, such as analytical statistics, and resources traditionally associated with the constructivist approach, such as qualitative data interpretation ^9^ .

The risks of a certain “disciplinary capture” must be recognized in the positivist models more traditionally associated with epidemiological, biomedical and clinical disciplines, which are prevalent in health research ^8^ . However, emerging evaluation models in Latin America seek to overcome the technocratic nature of the positivist aspects, incorporating qualitative-participatory dimensions as a restructuring axis of traditional models, focusing on the user-subject in the evaluation actions ^10 , 11^ .

We also recognize the possibility of working with quantitative data within a constructivist paradigm, provided that this information is not considered as “natural data revelations”. Alternatively, this data should be analyzed within the interpretative context of the agents involved in the research ^12 , 13^ .

Regarding the object of study, it is worth characterizing some relevant aspects of the Brazilian Unified Health System (SUS). The creation of a public and universal health system, instituted by the Federal Constitution of 1988, contributed to expand access to health and improve care for the population, positively affecting several indicators ^14^ . Brazil was one of the few large, middle-income countries to create a public health system of universal access.

Economic and political mishaps distorted the implementation of the system, preventing the equitable distribution of services between regions and municipalities. Traditionally, the South and Southeast regions have greater economic capacity and better access to health services, and the North and Northeast regions have lower per capita income, higher health needs and less access to services. Likewise, the state capitals have better installed capacity than other state municipalities ^15 , 16^ .

The SUS care network shows heterogeneities resulting from the history of health policies in Brazil, implying local differences in health priorities, resources and professionals’ allocation and funding models ^17^ . This variability makes evaluating the system even more challenging.

In Brazil, it was decided to constitute a system of territorial basis, whose priority gateway is PHC, with place of residence as catchment areas. PHC is organized according to the Family Health Strategy (FHS), which in turn presents different levels of implementation. The health care networks also are heterogeneous, with different coordination capacity of primary care ^18 , 19^ . These regional differences ^15 , 16^ are also observed among the various thematic care networks ^20 , 21^ .

The implementation of health care networks is still incipient, and often happens in the form of punctual, loco-regional and poorly standardized technological innovations, in pursuit of service integration ^22^ . This situation is aggravated by the country’s chronic regional inequality, as well as by the discontinuity of public policies after periodic changes of government officials. Evaluating the performance of care networks in this context requires the involvement of a wide variety of actors – sometimes for considerable periods.

### Diseases as Tracing Conditions

To analyze the access to specialized care via PHC, the performance of specialized care and its integration with other levels of complexity of the health system, we selected four tracing conditions ^25^ . This resource was developed in the 1970s to evaluate the quality of outpatient care and has been widely used ever since ^22 , 26 , 27^ . The principle of tracing conditions comes from choosing health problems with high prevalence, known evolution of the morbid process and clear identification of intervention measures, either manualized or not.

To select the tracing conditions, we used the following criteria: 1) chronic condition that requires diagnostic support for elucidation and with potential shared follow-up between PHC and specialized care; 2) peculiarities in care that generate the need for technologies available in different *loci* in the territory; 3) different dimensions of the health problem from user’s perception. Based on these criteria, we have chosen systemic arterial hypertension, high-risk pregnancy, breast cancer and severe mental disorders for our study.

The care pathways for systemic arterial hypertension and high-risk pregnancy are well defined, which allows a longitudinal follow-up of the provided care and the identification of a potential link with primary care in the process of comprehensive care. Severe systemic arterial hypertension also stands out due to its high prevalence and tendency to temporal increase. At-risk pregnancy does not represent a specific pathology, and pregnancy is a natural event of the course of life, with the possibility of complications that increase the risk of unfavorable outcomes for the woman and the unborn child. Both conditions are traditionally monitored in PHC, which confirms the influence of this level of care identifying escalation in case complexity to prompt other levels of care.

Breast cancer and severe mental disorders are diseases that often require increasing technologies (usually not available in PHC), due to the course of the condition and the associated suffering in the near social context. In the case of breast cancer, diagnosis, therapeutic support, professionals and specialized equipment are concentrated in tertiary services, which have a heterogeneous territorial distribution. Attention to severe mental disorders implies an intensification of the bond with the health team, the use of restricted medication as well as family and residential support, factors that require escalation of technology.

The adoption of these four tracers allowed a broad approach to the health system, so as not to restrict the analysis to a single specific program or network. We sought to address access to very diverse components of specialized care, having as common axis the investigation of the regulatory capacity of PHC.

### Components of the Multifaceted Methodology

The use of a linear approach in our research would contradict our understanding of the performance of care networks as a complex process that occurs in open systems ^13 , 28^ . Thus, we developed an analysis model based on mixed methods, with multiple data collection strategies, a wide variety of informants and an analysis plan that considers the tracing conditions and context specificities.

Initially, we conducted a study of secondary data, from which a series of indicators were elaborated, and simultaneously set forth a descriptive-qualitative study of the performance and regulation of the four studied cities’ networks. A first triangulation allowed us to know the SUS’ magnitude and evolution trend in each locality, serving as a context for the cross-sectional study performed sequentially. This first stage allowed us to formulate hypotheses about the use of health services in different contexts.

**Figure 1:**
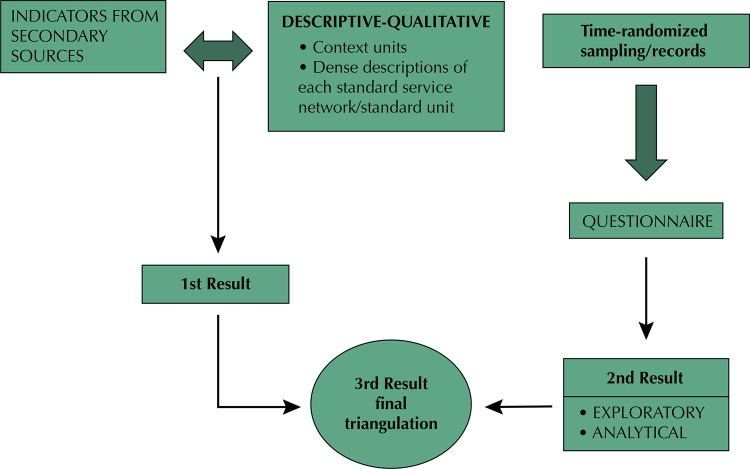
Analysis model stages.

The cross-sectional study included 7,053 users of specialized services from the four cities included in our study, seeking to identify these patients’ pathways, their access to specialized services and complementary exams and the presence of PHC for each disease. Additionally, we conducted qualitative studies focused on deepening the understanding of some results, when the analytical statistical analysis raised new questions that extrapolated the explanatory capacity of the quantitative results, as we address further in this paper. Figure 1 shows the multifaceted strategy.

### Secondary Data

We analyzed the characteristics, dimensions and temporal trends of PHC and specialized care by estimating ratios between dimensions (and trends), reference strategies between services and assessments of service flows. In this search, we used tab tools (database queries) made available by Datasus through Tabnet ^29^ and Tabwin ^30^ .

#### Time series studies

Time series have been widely used, since they allow a critical analysis of the directionality of the service supply, showing an increase or decrease in production, thus allowing estimations of access improvement or decrease. The study of the magnitude of the procedures or indicators constructed, in turn, allowed analyzing the relationships with the other procedures defined in the care pathway, as well as with the supply of health services structure and their regional differences. The analysis of the procedures magnitude, performed in each of the tracers allows us to infer how these procedures are being provided, considering the logical sequence predicted for each disease (ultrasonography, tomography, echocardiogram, etc.). This analysis, which reflects the availability of care resources, was interpreted considering the information gathered in the descriptive qualitative complement that we will describe below, constituting a first triangulation.

#### Data sources

We used the following official SUS databases: *Cadastro Nacional de Estabelecimentos de Saúde* (CNES – National Registry of Health Establishments), *Sistema de Informações sobre Nascidos Vivos* (SINASC – Live Birth Information System), *Sistema de Informação do Câncer da Mulher* (SISCAM – Women’s Cancer Information System), *Sistema de Informações Ambulatoriais* (SIA – Outpatient Information System), *Sistema de Informações Hospitalares* (SIH – Hospital Information System), and demographic data.

The demographic data were based on the population estimates provided by the Brazilian Institute of Geography and Statistics (IBGE), accessed by the Resident Population Estimates for the *Tribunal de Contas da União* (TCU – Federal Court of Accounts) from 2011 to 2014, available on Datasus according to the state ^29^ . The estimates by gender and age group, used to estimate some indicators, were obtained from these same sources.

For output indicators, based on SIA, we use the presented output, not just the frequency. The mental health data of Fortaleza were obtained through a form used by the city network, since the municipality did not have a computerized system for the *Registro das Ações Ambulatoriais de Saúde* (RAAS – Registry of Outpatient Health Actions). The information was made available by the municipality.

#### The design of indicators

Twenty-four indicators were design, divided into *structure indicators* (available resources, e.g., percentage of coverage), *access indicators* (management in the service-user interaction, e.g., number of consultations or hospitalizations per population), *effectiveness* indicators (scope and effects), and *service continuity and interaction indicators* (e.g., percentage of those referred by primary care). Box 1 shows the list of the 24 indicators, with calculation methods and data sources.

**Box 1:** Indicators for systemic arterial hypertension, at-risk pregnancy, breast cancer and mental health

Nº	Name of the indicator	Calculation method	Data source	Limitations of use
1	Type and number of units	Type of establishment, second model, management and etc.	TABNET (Ministry of Health)	
2	Coverage of primary care teams (FHS and equivalent)	$\frac{\left(N^{\circ }\text{ of }FHS+N^{\circ }\text{ Equivalent }FHS\right)\times 3,000}{\text{ Population of the same place and period }}\times 100$	SIA-SUS	Bias: numerator by occurrence and denominator per residence
3	Ratio for basic medical consultations (urgent and other)/population.	$\frac{\text{ Medical appointment by place of consultation }}{\text{ Total population (census and estimates) }}$	Sinasc	Bias: numerator by occurrence and denominator per residence
4	Proportion of prenatal care with seven or more consultations	Tabulate the number of prenatal consultations (seven or + consultations) for live births per mother’s residence, per year. After tabbing, perform ratio calculation for each range of number of queries.	SIA-SUS or equivalent	Bias: numerator by occurrence and denominator per residence
5	Ratio of the number of visits (individual and group sessions) performed by a psychiatrist in the PHC/population	$\frac{\mathrm{Appointment}\;\mathrm{with}\;a\;\mathrm{psychiatrist}\;(\mathrm{individual}\;\mathrm{and}\;\mathrm{group}\;\mathrm{sessions})\;\mathrm{in}\;\mathrm{PHC}\;\mathrm{by}\;\mathrm{place}\;\mathrm{and}\;\mathrm{year}}{\mathrm{Total}\;\mathrm{population}\;(\mathrm{census}\;\mathrm{and}\;\mathrm{estimates})\;\times \;10,000}$	SIA-SUS or equivalent	Bias: numerator by occurrence and denominator per residence
6	Ratio of the number of visits (individual and group sessions) performed by a psychologist in the PHC/population	$\frac{\mathrm{Appointment}\;\mathrm{with}\;a\;\mathrm{psychologist}\;(\mathrm{individual}\;\mathrm{and}\;\mathrm{group}\;\mathrm{sessions})\;\mathrm{in}\;\mathrm{PHC}\;\mathrm{by}\;\mathrm{place}\;\mathrm{and}\;\mathrm{year}}{\mathrm{Total}\;\mathrm{population}\;(\mathrm{census}\;\mathrm{and}\;\mathrm{estimates})\;\times \;10,000}$	SIA-SUS	Bias: numerator by occurrence and denominator per residence
7	Ratio for specialized consultation with a cardiologist/ population	$\frac{\mathrm{Number}\;\mathrm{of}\;\mathrm{appointments}\;\mathrm{with}\;a\;\mathrm{cardiologist}\;\mathrm{per}\;\mathrm{year}\;+\;\mathrm{cardiology}\;\mathrm{physician}\;+\;\mathrm{cardiologist},\;\mathrm{hearth}\;\mathrm{doctor}\;\mathrm{per}\;\mathrm{year}}{\mathrm{Total}\;\mathrm{population}\;\mathrm{per}\;\mathrm{year}\;\times \;1,000}$	SIA-SUS or equivalent	Bias: numerator by occurrence and denominator per residence
8	Echocardiogram ratio per 1,000 inhabitants/year	$\frac{\mathrm{Number}\;\mathrm{of}\;\mathrm{stress}\;\mathrm{echocardiographies}\;+\;\mathrm{transesophageal}\;\mathrm{echocardiographies}\;+\;\mathrm{transthoracic}\;\mathrm{echocardiographies}\;\mathrm{per}\;\mathrm{year}}{\mathrm{Total}\;\mathrm{population}\;\mathrm{per}\;\mathrm{year}\;\times \;1,000}$	AH-SIH	
9	Hospitalization ratio due to hypertension per 10,000 inhabitants	$\frac{\mathrm{Number}\;\mathrm{of}\;\mathrm{hospitalizations}\;\mathrm{with}\;\mathrm{primary}\;\mathrm{ICD}\;I10\;\mathrm{or}\;I11\;+\;\mathrm{Number}\;\mathrm{of}\;\mathrm{hospitalizations}\;\mathrm{with}\;\mathrm{secondary}\;\mathrm{ICD}\;I10\;\mathrm{or}\;I11}{\mathrm{Total}\;\mathrm{population}\;\mathrm{per}\;\mathrm{year}\;\times \;10,000}$	AIH-SIH	
10	Mammography ratio performed in the population aged 50 to 69 years ^a^	$\frac{\mathrm{Number}\;\mathrm{of}\;\mathrm{mammograms}\;+\;\mathrm{pre}-\mathrm{surgical}\;\mathrm{marking}\;\mathrm{of}\;a\;\mathrm{non}-\mathrm{palpable}\;\mathrm{lesion}\;\mathrm{in}\;\mathrm{the}\;\mathrm{mamma}\;\mathrm{corresponding}\;\mathrm{to}\;\mathrm{the}\;\mathrm{mammography}\;+\;\mathrm{tracing}\;\mathrm{bilateral}\;\mathrm{mammography}\;\mathrm{in}\;\mathrm{women}\;\mathrm{aged}\;\mathrm{between}\;30\;\mathrm{and}\;70\;\mathrm{years}\;\mathrm{per}\;\mathrm{year}}{\mathrm{Number}\;\mathrm{of}\;\mathrm{women}\;\mathrm{aged}\;\mathrm{between}\;30\;\mathrm{and}\;70\;\mathrm{years}\;\mathrm{per}\;\mathrm{year}}\;\times \;2$	SIA-PA and IBGE census and estimates	
11	Proportion of Cat mammography or more in the total number of mammograms performed	$\frac{\mathrm{Sum}\;\mathrm{of}\;\mathrm{the}\;\mathrm{number}\;\mathrm{of}\;\mathrm{CAT}\;\mathrm{four}\;\mathrm{to}\;\mathrm{six}\;\mathrm{mammographies}\;\mathrm{in}\;\mathrm{women}\;\mathrm{aged}\;50\;\mathrm{years}\;\mathrm{or}\;\mathrm{over}}{\mathrm{Total}\;\mathrm{number}\;\mathrm{of}\;\mathrm{CAT}\;\mathrm{zero}\;\mathrm{to}\;\mathrm{six}\;\mathrm{mammographies}\;\mathrm{in}\;\mathrm{women}\;\mathrm{aged}\;50\;\mathrm{years}\;\mathrm{or}\;\mathrm{over}\;\times \;100}$	Sismama/Siscan	
12	Ratio of hospitalization for breast cancer of women aged 50 years or over/female population aged 50 years and over	$\frac{\mathrm{Number}\;\mathrm{of}\;\mathrm{hospitalizations}\;\mathrm{of}\;\mathrm{women}\;\mathrm{aged}\;50\;\mathrm{years}\;\mathrm{or}\;\mathrm{over}\;\mathrm{with}\;\mathrm{ICD}\;10\;\mathrm{breast}\;\mathrm{malignant}\;\mathrm{neoplasm}\;\mathrm{per}\;\mathrm{year}}{\mathrm{Number}\;\mathrm{of}\;\mathrm{women}\;\mathrm{aged}\;50\;\mathrm{or}\;\mathrm{over}\;\times \;10,000}$	IBGE and SIH	
13	Ratio for specialized medical consultation with obstetrician/ live births	$\frac{Nº\;\mathrm{of}\;\mathrm{appointments}\;\mathrm{with}\;a\;\mathrm{gynecologist}\;\mathrm{and}\;\mathrm{obstetrician}\;+\;\mathrm{gynecologist}\;\mathrm{and}\;\mathrm{surgeon}\;\mathrm{gynecologist}\;\mathrm{obstetrician}\;+\;\mathrm{obstetrician}\;\mathrm{in}\;\mathrm{appointment}\;\mathrm{in}\;\mathrm{specialized}\;\mathrm{care}\;\mathrm{service}}{\mathrm{Number}\;\mathrm{of}\;\mathrm{live}\;\mathrm{births}\;\mathrm{per}\;\mathrm{year}}$	Siasus or equivalent and Sinasc	Biases: 1) confounding factor: added specialized consultations in gynecology and obstetrics 2) numerator by occurrence and denominator per residence
14	Ratio of Morphological obstetric US/live births	$\frac{\mathrm{Number}\;\mathrm{of}\;\mathrm{doppler}\;\mathrm{blood}\;\mathrm{flow}\;\mathrm{ultrasonographies}\;\mathrm{in}\;\mathrm{pregnancy}\;+\;\mathrm{obstetric}\;\mathrm{ultrasonographies}\;+\;\mathrm{obstetric}\;\mathrm{ultrasonographies}\;\mathrm{with}\;\mathrm{color}\;\mathrm{and}\;\mathrm{pulsed}\;\mathrm{doppler}\;\mathrm{per}\;\mathrm{year}}{\mathrm{Number}\;\mathrm{of}\;\mathrm{live}\;\mathrm{births}\;\mathrm{per}\;\mathrm{year}}$	Siasus or equivalent and Sinasc	Bias: numerator by occurrence and denominator per residence
15	Ratio of Number of deliveries in risk pregnancy/live births	$\frac{\mathrm{Number}\;\mathrm{of}\;\mathrm{normal}\;\mathrm{deliveries}\;\mathrm{in}\;\mathrm{risk}\;\mathrm{pregnancy}\;\mathrm{and}\;\mathrm{cesarean}\;\mathrm{sections}\;\mathrm{in}\;\mathrm{risk}\;\mathrm{pregnancy}\;\mathrm{per}\;\mathrm{year}}{\mathrm{Number}\;\mathrm{of}\;\mathrm{live}\;\mathrm{births}\;\mathrm{per}\;\mathrm{year}\;\times \;100}$	AH-SIH and Sinasc	
16	Apgar ratio greater than or equal to eight in the fifth minute/live births	$\frac{\mathrm{Live}\;\mathrm{births}\;\mathrm{by}\;\mathrm{mother}’s\;\mathrm{place}\;\mathrm{of}\;\mathrm{living},\;\mathrm{after}\;\mathrm{the}\;5\mathrm{th}\;\mathrm{minute}\;\mathrm{with}\;\mathrm{Apgar}\;\mathrm{score}\;8,\;9\;\mathrm{and}\;10\;\mathrm{per}\;\mathrm{year}}{\mathrm{Live}\;\mathrm{births}\;\mathrm{by}\;\mathrm{mother}’s\;\mathrm{place}\;\mathrm{of}\;\mathrm{living}\;\mathrm{per}\;\mathrm{year}}$	Source: Sinasc	
17	Average monthly number of caps registrations selected per 10,000 inhabitants (20 years and over) per year	$\frac{\mathrm{Number}\;\mathrm{of}\;\mathrm{patients}\;\mathrm{in}\;\mathrm{each}\;\mathrm{selected}\;\mathrm{Caps}\;(\mathrm{Caps}\;\mathrm{AD}\;\mathrm{and}\;\mathrm{Capsi}\;\mathrm{excluded})\;\mathrm{per}\;\mathrm{year}/\;12\;\mathrm{months}}{\mathrm{Estimated}\;\mathrm{population}\;\mathrm{aged}\;20\;\mathrm{years}\;\mathrm{and}\;\mathrm{over}\;\mathrm{in}\;\mathrm{that}\;\mathrm{year}\;\times \;10,000}$	SIA-RAAS	
18	Annual coverage of adult Caps by adult population	$\frac{\mathrm{Number}\;\mathrm{of}\;\mathrm{patients}\;\mathrm{in}\;\mathrm{each}\;\mathrm{selected}\;\mathrm{Caps}\;(\mathrm{Caps}\;\mathrm{AD}\;\mathrm{and}\;\mathrm{Capsi}\;\mathrm{excluded})\;\mathrm{per}\;\mathrm{year}}{\mathrm{Estimated}\;\mathrm{population}\;\mathrm{aged}\;20\;\mathrm{years}\;\mathrm{and}\;\mathrm{over}\;\mathrm{in}\;\mathrm{that}\;\mathrm{year}\;\times \;10,000}$	SIA-RAAS	
19	Selected procedure DAY TIME RECEPTION OF PATIENTS IN PSYCHOSOCIAL CARE CENTER per 10,000 inhabitants/year	$\frac{\mathrm{Number}\;\mathrm{daytime}\;\mathrm{reception}\;\mathrm{of}\;\mathrm{patients}\;\mathrm{in}\;\mathrm{each}\;\mathrm{selected}\;\mathrm{Caps}\;(\mathrm{Caps}\;\mathrm{AD}\;\mathrm{and}\;\mathrm{Capsi}\;\mathrm{excluded})}{\mathrm{Estimated}\;\mathrm{population}\;\mathrm{aged}\;20\;\mathrm{years}\;\mathrm{and}\;\mathrm{over}\;\mathrm{in}\;\mathrm{that}\;\mathrm{year}\;\times \;10,000}$	SIA-RAAS	Bias: numerator by occurrence and denominator per residence
20	PATIENT GROUP CARE IN PSYCHOSOCIAL CARE CENTER per 10,000 inhabitants/year in Caps	$\frac{\mathrm{Number}\;\mathrm{of}\;\mathrm{group}\;\mathrm{sessions}\;\mathrm{in}\;\mathrm{each}\;\mathrm{selected}\;\mathrm{Caps}\;(\mathrm{Caps}\;\mathrm{AD}\;\mathrm{and}\;\mathrm{Capsi}\;\mathrm{excluded})}{\mathrm{Estimated}\;\mathrm{population}\;\mathrm{aged}\;20\;\mathrm{years}\;\mathrm{and}\;\mathrm{over}\;\mathrm{in}\;\mathrm{that}\;\mathrm{year}\;\times \;10,000}$	SIA-RAAS	Bias: numerator by occurrence and denominator per residence
21	Selected procedure INDIVIDUAL PATIENT CARE IN PSYCHOSOCIAL CARE CENTER per 10,000 inhabitants/year in Caps	$\frac{\mathrm{Number}\;\mathrm{of}\;\mathrm{individual}\;\mathrm{sessions}\;\mathrm{in}\;\mathrm{each}\;\mathrm{selected}\;\mathrm{Caps}\;(\mathrm{Caps}\;\mathrm{AD}\;\mathrm{and}\;\mathrm{Capsi}\;\mathrm{excluded})}{\mathrm{Estimated}\;\mathrm{population}\;\mathrm{aged}\;20\;\mathrm{years}\;\mathrm{and}\;\mathrm{over}\;\mathrm{in}\;\mathrm{that}\;\mathrm{year}\;\times \;10,000}$	SIA-RAAS	Bias: numerator by occurrence and denominator per residence
22	Proportion of patients that came from primary care	$\frac{\mathrm{Average}\;\mathrm{number}\;\mathrm{of}\;\mathrm{patients}\;\mathrm{per}\;\mathrm{month}\;\mathrm{referred}\;\mathrm{from}\;\mathrm{PHC}}{\mathrm{Average}\;\mathrm{number}\;\mathrm{of}\;\mathrm{patients}\;\mathrm{per}\;\mathrm{month}}$	SIA-RAAS	
23	Ratio of psychiatric hospitalizations SUS/ population (number of monthly invoices of AH per 1,000 inhabitants/year)	$\frac{\mathrm{Number}\;\mathrm{of}\;\mathrm{psychiatric}\;\mathrm{hospitalizations}\;\mathrm{per}\;\mathrm{year}}{\mathrm{Total}\;\mathrm{population}\;\mathrm{in}\;\mathrm{that}\;\mathrm{year}\;\times \;1,000}$	AH-SIH and CNES	
24	Ratio for SUS hospitalizations in general hospital/ SUS hospitalizations in psychiatric hospital ^b^	$\frac{\mathrm{Hospitalizations}\;\mathrm{in}\;\mathrm{general}\;\mathrm{hospitals}}{\mathrm{Hospitalizations}\;\mathrm{in}\;\mathrm{psychiatric}\;\mathrm{hospitals}}$	AH-SIH and CNES	

^a^ Result value × 2, because the parameter is an exam conducted every two years.

^b^ Use of Indicator 23.

PC: primary care; AH: authorization of hospitalization; CAPS: psychosocial care center; CAPS AD: CAPS alcohol and other drugs; CAPSi: CAPS children and young people; CAT4: category 4; ICD: International Classification of Diseases; CNES: *Cadastro Nacional de Estabelecimentos de Saúde* (National Registry of Health Establishments); FHS: Family Health Strategy; IBGE: Brazilian Institute of Geography and Statistics; RAAS: *Registro das Ações Ambulatoriais de Saúde* (Registry of Outpatient Health Actions); SIA-SUS: *Sistema de Informações Ambulatoriais do SUS* (SUS Outpatient Information System); SIH: *Sistema de Informações Hospitalares* (Hospital Information System); SINASC: *Sistema de Informações sobre Nascidos Vivos* (Live Birth Information System); SISCAN: *Sistema de Informação do Câncer* (Cancer Information System); SISMAMA: *Sistema de Informação do câncer de mama* (Breast cancer information system); SUS: Unified Health System; US: ultrasonography.

In this initial approach of secondary data, we were able to identify differences in each municipality’s service networks, for each specific disease. Critical analysis of the relation between the different procedures expected in each care pathway allowed differentiating the type of supply and the role of care regulation in each municipality’s health issues.

## The Qualitative-Descriptive Complement

A descriptive-qualitative study was conducted for each city, seeking a dense description of the service network for each health condition, performance singularities and most relevant historical events. For such purpose, a standard script was constructed, collectively validated by the research team. Each of the script items was filled out by a group of researchers from each locality, based on the available sources and using key informants when necessary.

The listed questions included: information on primary care performance; Family Health Support Centers (FHSC) and mental health support teams availability and composition; model of scheduling specialized consultations; use of referral/counter-referral; organization patterns for each thematic healthcare network; availability of university services (either integrated with SUS, or not); and model of beds regulation for relevant clinics to each disease. Box 2 shows the information contained in the script for each health condition and city.

**Box 2:** Qualitative schema information

Public and affiliated network
Performance of primary care (FHS, mixed, programmatic, emergency care, form of connection of complications, use of risk assessment with or without reception)
Thematic network (is there a thematic network for this disease? how the initial access and traffic to other points of care occur?)
What specialties offer matrix/FHSC support?
Are there university services (hospital, emergency care)? Is there any regulation of vacancies by the city?
Description of the query scheduling center
Description of the vacancy control plant
Other unique features of the local network and its history that deserve to be detailed

FHS: Family Health Strategy; FHSC: Family Health Support Center.

This qualitative information allowed to further understand each local context and informed a complementary analysis of magnitudes and trends evidenced by the information systems indicators, constituting a first round of triangulation.

## Service Survey: Drawing, Sampling, Analysis Plan

For the cross-sectional study with users of the referenced health services, the following types of services were chosen:

- Breast cancer: oncology high complexity centers.
- Mental health: Psychosocial Care Centers (CAPS);
- Hypertension: cardiology outpatient clinics.
- Risky pregnancy: maternity wards, obstetric outpatient clinics.

We chose specialized services to ensure greater representativeness of each city’s health network: it becomes possible to approach all specialized services, since they are few, but receive primary care users from the entire municipality. Thus, we developed four questionnaires addressing aspects of PHC and specialized care related to each disease and applied them to users under follow-up in specialized care.

The questionnaires for each disease, containing between 49 and 66 questions, aimed to identify and measure events related to the users’ trajectory in primary and specialized care services. These events were fundamentally good practices based on protocols or the scientific literature of the area, such as interventions and conducts, as well as their waiting times, frequency and place (PHC or specialized care) of occurrence.

The four questionnaires share six axes that guide the questions: 1) sociodemographic information; 2) characteristics of specialized health care; 3) characteristics of primary care; 4) medication, complementary exams and orientations, especially in the primary-specialized care path; 5) use of urgency and emergency services; and 6) use of health plans and paid health services. The full version is available on the research website.

### Sampling

Different sampling approaches were used according to the particularities of each municipality and disease. A total of 7,053 users were interviewed, divided into the four tracing conditions. Box 3 shows the strategies used. Detailed approach to randomization methods can be found on the research website ^a^ .

**Box 3:** Sampling strategies for each health condition and municipality.

Municipality	Disease	Type of sample	Services in the municipality	Participating search services	Total surveys
Campinas	Hypertension	Simple sample	4	4	485
Fortaleza	Hypertension	Simple sample	5	5	417
Porto Alegre	Hypertension	Simple sample	9	4	408
São Paulo	Hypertension	Two-stage cluster sampling (services and users)	52	30	760
Campinas	Risk pregnancy	Simple sample	3	3	500
Fortaleza	Risk pregnancy	Simple sample	5	5	401
Porto Alegre	Risk pregnancy	Simple sample	5	5	391
São Paulo	Risk pregnancy	Simple sample	15	15	689
Campinas	Breast cancer	Simple sample	3	2	318
Fortaleza	Breast cancer	Simple sample	5	5	334
Porto Alegre	Breast cancer	Simple sample	5	5	355
São Paulo	Breast cancer	Simple sample	4	1	353
Campinas	Mental health	Census	6	6	393
Fortaleza	Mental health	Census	6	6	601
Porto Alegre	Mental health	Census	4	4	351
São Paulo	Mental health	Simple sample	34	24	297

### Analysis Plan

The first analysis of the data from the questionnaires aimed to identify significant differences in the recollected events. We compared these differences with sociodemographic characteristics to allow a more adequate analysis of significant relationships. Complementing these bivariate analyses, we performed multivariate analyses to control intervening or correlated variables. We sought to analyze these associations including the following dimensions: access, bonding and care practices.

At that moment, some questions for each health condition could be clarified by a new crossing with the indicators from secondary data (second triangulation).

### The Qualitative-Descriptive Complement

After this sequence of procedures, some qualitative-design focuses were explored to increase the capacity of analysis when the data from the previous stages demanded deeper probing.

In-depth interviews were performed with key actors, records of users’ therapeutic itineraries, life history narratives and active search for user losses from specialized services, according to each case. These focuses of qualitative studies allowed us to progress on some questions, interpret incomprehensible correlations and increase the design’s analytical power.

## DISCUSSION

We argue that the multifaceted design and mixed methods allowed an interesting approximation to the real complexity of the Brazilian health system, providing an explanatory analysis through the sequential and simultaneous combination of complementary approaches.

The study relies on quantitative data and many interviewees. The sequential phases of analysis allowed a long-term engagement of the researchers and repeated data audits and reinterpretations. Qualitative information enriched the interpretation of statistical analyses, even qualifying some unclear findings of the service survey. This triangulation of methods allowed to expand and advance the investigation of the discussed themes, a strategy that is still uncommon in health policies and systems studies, although recommended in the literature ^4 , 5 , 8^ . We point out below some specific points that indicate the advantages of this triangulation.

The sequence of stages in the analysis plan provided greater sensitivity regarding the regional inequalities and each cities’ specificities. The initial assumption of each municipality as an autonomous case study allowed us to understand local history and context, unveiling sudden changes in important indicators in certain cities, in specific years, as well as historical priorities in the development of certain health care models.

Regarding the indicators sensibility to local administrations’ priorities, we identified changes in PHC coverage, in the proportion between traditional PHC and FHS, and in the provision of FHSC teams. Stability or oscillations were also verified in specific procedures of each tracing condition, in PHC and/or in specialized care.

The interviews with key informants, that produced the qualitative-descriptive mask, allowed us to approach other actors’ voices, engaged in management and administration level of services, a procedure whose importance has already been emphasized in other studies ^3^ .

This set of information qualified the perceptions about the specific contexts, from which the access to specialized care occurred through the PHC of each municipality, for each health condition. In short, the time series of secondary data and indicators contributed to historically frame the results of the cross-sectional study and the qualitative component. The qualitative interviews, in turn, complemented the statistical analysis of the cross-sectional study in a new round of the data analysis spiral.

Even with a predominant quantitative component in our research, we consider that the information produced is closer to the “qualitative” model of theoretical generalization ^7^ . Based on it, the comparison between the findings of each health condition, in each municipality, allowed us to find cross-sectional regularities in the different focuses of analysis (health condition and municipality), but also isolated effects of specific *loci.*

The need to contextualize our findings created a different task from that of traditional epidemiological studies – whose main concern is to isolate variables that have significant effects on other variables – since it would not be possible to isolate our variables of interest outside the context of each network and municipality due to our interest in understanding such singular performances.

Traditionally, the concern with the context of the findings is manifested by interpretations of the culturally attributed meanings to a given phenomenon. Although this conception was present in our qualitative component, we emphasize that the characterization of each municipality’s context was also analyzed in the light of the descriptive and analytical statistics performed in the indicators and in the results of the in-service surveys.

The literature points out that the difficulties arising from reducing relevant contextual factors to a set of quantifiable measures have generated dense descriptions, common in anthropological references ^8^ . However, our triangulation allowed us to approach the context through two distinct aspects, including in the analysis the particular experiences of the people involved, resulting in dense descriptions, in conjunction with objective elements measured quantitatively, thus creating different “layers” of context.

We emphasize, for example, associations between variables that occurred only in some of the cities investigated, which allowed the identification of the different performances of each local network mode of operation, issues to be further assimilated in the qualitative component.

The capacity of complete generalization of scientific evidence has been questioned. Systematic reviews of randomized clinical trials have brought conflicting information on the effectiveness of interventions, pointing out that certain actions, which demonstrate effects in a specific research or implementation scenario, do not necessarily present the same results in a different scenario ^5 , 31^ .

Although understanding subjectivity is one of the central elements of qualitative research, the use of patient-reported outcomes has also been advocated in clinical research, from quantitative epistemology, especially when the results to be measured do not allow direct observation ^32 , 33^ . The importance of a series of complex phenomena has been recognized, which can only be known from the patient’s own report, by either interviews, questionnaires or scales, to comparatively capture the perception of the object studied from different interest groups.

In accordance with these premises, we developed a survey with users of specialized services, incorporating the advantages of this approach, but recognizing its limitations. The main advantage was to allow the possibility of analyses intra-service, in between services and in between municipalities, for each health condition investigated, respecting the different levels of statistical significance in each comparative analysis.

Regarding the limitations of the cross-sectional survey, both overestimation and underestimation of service use in self-reported questionnaires were observed in previous studies ^32^ . Events recalled after 12 months and recurrent events tend to be more often underestimated; rare and striking events, such as hospitalizations, present little memory bias. There is also a variation between the information of self-reported mental health diagnoses and medical records ^34^ , a bias that may also have influenced our study.

Despite these limitations, we consider that the service users’ survey can produce irreplaceable information, helping to correct weaknesses resulting from under-notification in information systems, as well as addressing precise details not cataloged by these systems.

## FINAL REMARKS

The multifaceted analytical model presented allowed us to understand the performance of a universal public health system in a middle-income country, considering the singularities, heterogeneities and inequalities that characterize the implementation of this system in Brazil.

The main characteristics of this construct were the use of tracing conditions to direct the analysis plan, having as guidelines the specific care pathways, as well as the use of the available secondary data, allowing the approximation to time series and the preliminary formulation of relationship hypotheses.

We also point out the mixed methods approach, which allowed us to incorporate a large number and wide range of interviewees at different levels of the system, with recurrent involvement with stakeholders, allowing to verify data and reinterpret the analyses when necessary. The qualitative approach allowed the identification of local variables that favored or hindered longitudinal care and regulation of the service network by PHC in the studied health problems.

The comprehensive geographical scope and the diversity of sites studied allowed us to analyze the performance of the SUS regarding the standards of good practices for the health conditions approached, although without claiming representativeness and generalization throughout Brazil.

Finally, the use of a multifaceted framework of research techniques allowed us to understand several dimensions of access to care, with sensitivity to detect formats excessively centered on specialists, contextualizing the information available in information systems.

We hope that this design will contribute to other studies that address the variability of SUS’ implementation in the country. The design of a simultaneous and sequential quantitative-qualitative approach, using tracing conditions, increased the analytical capacity of the approach, inserted several informants and allowed temporal analyses. By contemplating heterogeneities and particularities, this design also provided a broader understanding of the performance of local networks for the addressed health conditions. We consider that these points contain the main contributions of the presented research strategy for health systems analysis.
