# Supplementary material for: Notes for the study on health systems: multifaceted analysis and tracer indicators
Source: Rev Saude Publica. 2020 Oct 28;54:102. doi: 10.11606/s1518-8787.2020054002055 (PMC7593041; doi:10.11606/s1518-8787.2020054002055)
Supplement: Translation

# Translation: Apontamentos para estudo de sistemas de saúde: análise multifacetada e agravos traçadores

**Authors:** Rosana Teresa Onocko-Campos, Gastão Wagner de Sousa Campos, Carlos Eduardo Menezes Amaral, Oswaldo Yoshimi Tanaka

**Affiliations:** I Brasil Universidade Estadual de Campinas. Faculdade de Ciências Médicas. Departamento de Saúde Coletiva. Campinas, SP, Brasil; II Brasil Universidade Federal do Vale do São Francisco. Colegiado de Medicina de Paulo Afonso. Paulo Afonso, BA, Brasil; III Brasil Universidade de São Paulo. Faculdade de Saúde Pública. São Paulo, SP, Brasil

**Keywords:** Atenção Primária à Saúde, Atenção Secundária à Saúde, Triangulação de Métodos, Avaliação de Processos e Resultados em Cuidados de Saúde

## Abstract

**OBJETIVO:**

Apresentar a abordagem metodológica utilizada em pesquisa que analisou a utilização e o funcionamento da atenção especializada, partindo do acesso via atenção básica, em quatro grandes cidades brasileiras: Fortaleza (CE), Campinas (SP), São Paulo (SP) e Porto Alegre (RS).

**MÉTODOS:**

Apresentação e discussão dos componentes quanti-qualitativos da estratégia de pesquisa proposta.

**RESULTADOS:**

Foram estudadas quatro condições traçadoras: hipertensão arterial grave, gravidez de alto risco, câncer de mama e transtorno mental grave. Para cada agravo foram construídos indicadores a partir de dados dos sistemas de informação de saúde destacando frequências, tendência temporal e diferenças por localidade. Essa contextualização inicial foi enriquecida com um estudo descritivo-qualitativo do funcionamento de cada rede municipal de serviços. A seguir, realizou-se um estudo transversal por meio de inquérito com 7.053 usuários dos serviços especializados para cada agravo. Por fim, foram realizadas entrevistas em profundidade com atores-chave para complementar aspectos operacionais selecionados da rede de cada município. Os resultados de todas essas fontes de dados foram triangulados, permitindo explorar a variabilidade das implantações do SUS em diferentes cenários regionais.

**CONCLUSÕES:**

O modelo analítico multifacetado apresentado permite compreender aspectos relevantes do funcionamento do Sistema Único de Saúde, atentando para as singularidades, heterogeneidades e desigualdades que caracterizam sua implantação no Brasil e destacando o funcionamento das redes locais para os agravos estudados.

## INTRODUÇÃO

Neste artigo apresentamos uma combinação de métodos mistos e multifacetados desenvolvida por um grupo multicêntrico de investigadores brasileiros para analisar o funcionamento e a utilização da atenção especializada, partindo do acesso à atenção primária em saúde (APS), para quatro condições traçadoras em quatro grandes cidades brasileiras: Fortaleza (CE), Campinas (SP), São Paulo (SP) e Porto Alegre (RS).

Segundo a Organização Mundial da Saúde, o campo da análise de sistemas de saúde continua relativamente pouco explorado, particularmente nos países de baixa e média renda ^1^ . Na América Latina, a produção científica brasileira no campo de avaliação em saúde se destaca, com especial interesse na integração de serviços ocorrida após a implantação governamental de políticas de redes de atenção à saúde (RAS) na última década. Contudo, tanto na literatura brasileira como internacional, as pesquisas no campo de políticas e serviços de saúde tendem a focar programas e ações específicas em vez de sistemas nacionais ou locais de saúde ^2^ .

As análises de sistemas de saúde, quando ocorrem, têm se baseado em abordagens diversas. Todavia, revisão recente apontou como problema importante no conjunto de estudos sobre sistemas e serviços a excessiva confiança em métodos descritivos e estudos transversais ^3^ . Entre outros aspectos a serem superados em prol da acurácia e sofisticação do campo, a revisão aponta que a maioria dos estudos inclui um único tipo de informante ou nível de produção de informações, e que grande parte dos estudos utiliza dados de um único ponto temporal. No que diz respeito aos referenciais de análise, muitos trabalhos utilizam apenas a estatística descritiva, e a grande maioria não declara adequadamente seu nível de sensibilidade ou poder estatístico.

Outros autores também têm chamado a atenção para a necessidade de pesquisadores desenvolverem novas formas de análise, de modo a desenvolver uma lente adequada para representar e explicar cenários altamente complexos ^4^ . Os efeitos produzidos pelos sistemas de saúde não podem ser explicados por meio de uma relação de causalidade linear simples, sendo necessários referenciais que adotem a noção de causalidade complexa, entendida como causas múltiplas que interagem e geram efeitos por vezes imprevisíveis ^5^ .

A exigência de adotar a causalidade complexa deriva também da constatação de que ações e políticas com frequência não geram os mesmos impactos em lugares e tempos diferentes ^5^ . Por conseguinte, impõe-se à investigação dos sistemas de saúde a compreensão da história e contexto local dos subsistemas, uma vez que:

[...] quando a análise se desloca do cuidado médico para níveis mais complexos de organização das práticas de saúde em municípios ou distritos sanitários [...] o obscurecimento do contexto e da historicidade do objeto, operado pela suposta universidade do paradigma, compromete a compreensão do significado dos eventos. ^6^

Outro desafio às pesquisas de sistemas de saúde, relacionado às premissas anteriores, consiste na possibilidade de generalizar os achados. Tendo em vista a heterogeneidade dos sistemas nacionais de saúde e seus subsistemas locais, aliada à causalidade complexa que organiza seus efeitos, torna-se desafiador produzir conclusões de aplicação direta no conjunto genérico de sistemas e subsistemas de saúde.

Frente a isso, cabe destacar que os estudos devem buscar a generalização analítica. Esse tipo de generalização permite desenvolver conclusões gerais que, embora derivadas de experiências singulares, provêm insights teóricos a serem testados em outros contextos, orientando tanto pesquisas futuras como processos de tomada de decisão ^7 , 8^ .

Com base nessa caracterização, este trabalho busca apresentar um modelo analítico multifacetado, desenvolvido para investigar o funcionamento da atenção especializada e o acesso via APS em quatro grandes cidades brasileiras a partir de agravos traçadores.

## APRESENTAÇÃO DA ESTRATÉGIA DE PESQUISA

Buscamos com esse artigo apresentar uma estratégia de pesquisa condizente com os desafios que se apresentam ao campo, compartilhando a vivência de pesquisadores de diversas universidades, formuladores de políticas e profissionais de saúde de diferentes estados do Brasil.

Apresentamos nossa experiência como uma estratégia de pesquisa, entendendo-a não apenas como desenho de estudo ou método particular, mas como forma de se aproximar do sistema de saúde conjugando métodos de coleta de dados e amostragem da forma mais adequada possível aos propósitos e objeto de nossa investigação ^5^ . Cabe apontar, portanto, algumas premissas teórico-metodológicas da estratégia de pesquisa em relação a seus propósitos e objeto.

No que diz respeito aos propósitos da investigação, pode-se argumentar que, de maneira geral, as pesquisas sobre sistemas e políticas de saúde buscam aferir a qualidade desses dispositivos. Contudo, tal intenção traz consigo alguns complicadores. Dentre eles, destaca-se a pluralidade teórica de referenciais avaliativos, cuja distinção inicia-se na perspectiva ontológica e epistemológica adotada, o que traz implicações metodológicas importantes.

No campo da avaliação em saúde destacam-se, de modo geral, correntes em duas polaridades ontológicas e epistemológicas distintas: a perspectiva positivista e as abordagens interacionistas/construtivistas. Em nossa estratégia de pesquisa, reconhecemos a possibilidade de abreviar a distância entre as ferramentas do método positivista, tais como a estatística analítica e os dispositivos tradicionalmente associados à vertente construtivista, como a interpretação de dados qualitativos ^9^ .

Há de se reconhecer os riscos de certa “captura disciplinar” a partir dos modelos positivistas mais tradicionalmente associados às disciplinas epidemiológicas, biomédicas e clínicas, dominantes na pesquisa em saúde ^8^ . Todavia, há na América Latina modelos emergentes de avaliação que buscam superar o caráter tecnocrático das vertentes positivistas, incorporando dimensões qualitativo-participativas como eixo reestruturante dos modelos tradicionais, trazendo o sujeito-usuário como foco das ações avaliativas ^10 , 11^ .

Reconhecemos também a possibilidade de trabalhar com informações quantitativas dentro de um paradigma construtivista, contanto que essas informações não sejam consideradas “revelações de dados naturais”, sendo, alternativamente, analisadas dentro do contexto interpretativo do conjunto de agentes envolvidos na pesquisa ^12 , 13^ .

Em relação ao objeto de estudo, cabe caracterizar alguns aspectos relevantes do Sistema Único de Saúde (SUS). A criação de um sistema de saúde público e universal, a partir da Constituição de 1988, contribuiu para expandir o acesso à saúde e melhorar a assistência da população, impactando positivamente diversos indicadores ^14^ . O Brasil foi um dos poucos países de grandes dimensões e renda média a ter tomado a decisão de criar um sistema público de acesso universal.

Percalços econômicos e políticos trouxeram distorções na implantação do sistema, impedindo a distribuição equitativa de serviços entre as regiões e municípios. Tradicionalmente, as regiões Sul e Sudeste contam com maior capacidade econômica e melhor acesso aos serviços de saúde, e as regiões Norte e Nordeste com menor renda per capita, maiores necessidades de saúde e menor acesso a serviços. De forma semelhante, em geral, as capitais apresentam melhor capacidade instalada que os municípios do interior ^15 , 16^ .

A rede assistencial do SUS demonstra heterogeneidades decorrentes da história das políticas de saúde no Brasil, implicando diferenças locais nas prioridades sanitárias, na alocação de recursos e de profissionais e nos modelos de financiamento ^17^ . Essa variabilidade torna a avaliação do sistema ainda mais desafiadora.

No Brasil, decidiu-se por constituir um sistema de base territorial, cuja porta de entrada prioritária é a APS, com adscrição da população por área de residência. A APS se organiza conforme a Estratégia Saúde da Família (ESF), que apresenta diferentes níveis de implantação. Nas Redes de Atenção à Saúde, também há grande heterogeneidade, além de considerável variação na capacidade de coordenação por parte da atenção primária ^18 , 19^ . Essas diferenças regionais ^15 , 16^ também são observadas entre as diversas redes temáticas de atenção ^20 , 21^ .

A implantação das Redes de Atenção à Saúde é ainda incipiente, e muitas vezes acontece sob o formato de inovações tecnológicas pontuais, locorregionais e pouco normatizadas, na busca pela integração de serviços ^22^ . Essa situação é agravada pela crônica desigualdade regional brasileira, assim como pela descontinuidade das políticas públicas com as mudanças periódicas na gestão. Avaliar o funcionamento das redes assistenciais nesse contexto requer o envolvimento de uma grande variedade de atores – às vezes por períodos consideráveis.

### Agravos como Condições Traçadoras

Para analisar o acesso à atenção especializada via APS, assim como o funcionamento da atenção especializada e sua integração com outros níveis de complexidade do sistema de saúde, foram selecionadas quatro condições traçadoras ^25^ . Este recurso foi desenvolvido na década de 1970 para avaliar a qualidade do atendimento ambulatorial e tem sido amplamente utilizado desde então ^22 , 26 , 27^ . O princípio das condições traçadoras parte da escolha de agravos de saúde que tenham alta prevalência, evolução conhecida do processo mórbido e clara identificação de medidas de intervenção, protocolizadas ou não.

Para selecionar as condições traçadoras, utilizaram-se os seguintes critérios: 1) condição crônica que necessita de apoio diagnóstico para elucidação e com potencial de acompanhamento compartilhado entre APS e atenção especializada; 2) peculiaridades no cuidado que gerem necessidade de tecnologias disponíveis em distintos *loci* no território; 3) distintas dimensões do problema de saúde na percepção dos usuários. Com base nesses critérios foram escolhidos para o estudo os agravos hipertensão arterial sistêmica, gravidez de alto risco, câncer de mama e transtornos mentais graves.

As linhas de cuidado da hipertensão arterial sistêmica e da gestação de alto risco são bem definidas, o que permite o acompanhamento longitudinal do cuidado e a identificação de potencial vínculo com a atenção básica no processo de atenção integral. A hipertensão arterial sistêmica grave destaca-se também por sua alta prevalência e tendência de aumento temporal. Gravidez de risco não representa uma patologia específica, sendo a gestação um evento natural do curso da vida, com possibilidade de complicações que aumentam o risco de desfechos desfavoráveis à mulher e ao nascituro. Ambos os agravos são tradicionalmente acompanhados na APS, o que torna marcante a influência desse nível de atenção na identificação do aumento de complexidade dos casos para acionamento dos outros níveis de atenção.

O câncer de mama e os transtornos mentais graves são agravos que, pelo tipo de evolução e sofrimento que ocasionam no contexto social próximo, com frequência demandam tecnologias crescentes, geralmente não disponíveis na APS. No caso do câncer de mama, o diagnóstico, suporte terapêutico, profissionais e equipamentos especializados concentram-se em serviços terciários, com distribuição territorial heterogênea. A atenção ao transtorno mental grave implica intensificação do vínculo com a equipe de saúde, utilização de medicamentos de dispensação restrita e suporte familiar e domiciliar, fatores que demandam o escalonamento de tecnologia.

A adoção desses quatro traçadores permitiu uma abordagem ampla do sistema de saúde, de forma a não restringir a análise a apenas um programa ou rede específica. Buscou-se abordar o acesso a componentes bastante diversificados da atenção especializada, tendo como eixo comum a investigação da capacidade de regulação da APS.

### Componentes da Metodologia Multifacetada

O uso de uma abordagem linear em nossa pesquisa contradiria a compreensão do funcionamento das redes assistenciais como processo complexo que ocorre em sistemas abertos ^13 , 28^ . Assim, desenvolvemos neste estudo um modelo de análise baseado em métodos mistos, com múltiplas estratégias de coleta de dados, ampla variedade de informantes e plano de análise que considera as condições traçadoras e as especificidades do contexto.

Inicialmente, realizamos um estudo de dados secundários a partir dos quais foi elaborada uma série de indicadores, concomitante a um estudo descritivo-qualitativo do funcionamento e regulação das redes das quatro cidades estudadas. Assim, uma primeira triangulação nos permitiu conhecer a magnitude e a tendência de evolução do SUS em cada localidade, servindo de contexto para o estudo transversal realizado em seguida, de maneira sequencial. Essa primeira etapa permitiu formular hipóteses sobre o uso de serviços nos distintos contextos.

O estudo transversal incluiu 7.053 usuários de serviços especializados das quatro cidades incluídas no estudo, buscando identificar a trajetória desses pacientes, o acesso aos serviços especializados e a exames complementares e a vinculação à APS para cada agravo. Adicionalmente, realizamos estudos qualitativos focados em aprofundar a compreensão de alguns resultados, quando a análise estatística analítica levantou novas questões que extrapolavam a capacidade explicativa dos resultados quantitativos, conforme desenvolveremos a seguir. Na Figura 1 resumimos a estratégia multifacetada apresentada.

**Figura 1:** Fases do modelo de análise.

### Dados Secundários

Foram analisadas características, dimensões e tendências temporais da APS e atenção especializada a partir da construção de razões entre as dimensões (e tendências), estratégias de referência entre os serviços e levantamentos dos fluxos de atendimento. Utilizamos nesta pesquisa ferramentas de tabulação (consultas a bases de dados) disponibilizadas pelo Datasus por meio do Tabnet ^29^ e Tabwin ^30^ .

#### Estudos de séries temporais

As séries temporais têm sido muito utilizadas, pois permitem uma análise crítica da direcionalidade da oferta de serviços, mostrando aumento ou diminuição da produção, permitindo, assim, estimar melhorias ou pioras no acesso. Por sua vez, o estudo da magnitude dos procedimentos ou dos indicadores construídos permitiu analisar as relações com os outros procedimentos definidos na linha de cuidado, bem como com a oferta estrutural dos serviços de saúde e suas diferenças regionais. A análise da magnitude dos procedimentos realizados em cada uma das traçadoras permite inferir como estas estão sendo providas, tendo em vista a sequência lógica prevista para cada agravo (ultrassonografia, tomografia, ecocardiograma etc.). Essa análise, que reflete a disponibilidade dos recursos assistenciais, foi interpretada à luz das informações levantadas no complemento qualitativo descritivo que descreveremos a seguir, constituindo uma primeira triangulação.

#### Fontes de dados

Utilizamos como fonte as bases de dados oficiais do SUS: Cadastro Nacional de Estabelecimentos de Saúde (CNES), Sistema de Informações de Nascidos Vivos (Sinasc), Sistema de Informação do Câncer da Mulher (Siscam), Sistema de Informações Ambulatoriais (SIA), Sistema de Informações Hospitalares (SIH) e dados demográficos.

Para os dados demográficos, as estimativas foram feitas a partir das estimativas de população fornecidas pelo Instituto Brasileiro de Geografia e Estatística (IBGE), acessadas pelas Estimativas de População Residente para o TCU de 2011 a 2014, disponibilizadas no Datasus de acordo com o estado ^29^ . As estimativas por sexo e faixa etária, utilizadas para o cálculo de alguns indicadores, foram obtidas nessas mesmas fontes.

Para os indicadores de produção, baseados no SIA, utilizamos a produção apresentada, e não apenas a frequência. Os dados de saúde mental de Fortaleza foram obtidos através de um formulário utilizado pela rede da cidade, uma vez que o município não dispunha de sistema informatizado para o Registro das Ações Ambulatoriais de Saúde (RAAS). As informações foram disponibilizadas pelo próprio município.

#### A construção dos indicadores

Foram construídos 24 indicadores, divididos entre indicadores de *estrutura* (recursos disponíveis, p. ex., percentual de cobertura), *acesso* (manejo na interação serviço-usuário, p. ex., número de consultas ou internações por população), *efetividade* (alcance e efeitos) e *continuidade e interação entre serviços* (p. ex., percentual de encaminhados pela atenção básica. A lista dos 24 indicadores, com método de cálculo e fonte de dados, encontra-se no Quadro 1 .

**Quadro 1:** Indicadores para hipertensão arterial sistêmica, gestação de risco, câncer de mama e saúde mental

Nº	Nome do indicador	Método de cálculo	Fonte de dados	Limitações de uso
1	Tipo e número de unidades	Tabular tipo de estabelecimento, segundo modelo, gestão e etc	CNES e cadastros específicos	
2	Cobertura de equipes da atenção básica (ESF e equivalentes)	$$\frac{(N^{\circ }deESF+N^{\circ }\text{ de ESF equivalente) }x3.000}{\text{ População no mesmo local e periodo }}\times 100$$	TABNET (Ministério da Saúde)	
3	Razão de consultas médicas básicas (de urgência e demais)/população.	$$\frac{\text{ Consulta médica na AB por local de atendimento }}{\text{ População total (censo e estimativas) }}$$	SIA-SUS	Viés: numerador por ocorrência e denominador por residência
4	Proporção de pré-natal com sete ou mais consultas	Tabular o número de consultas de pré-natal (sete ou + consultas) por nascidos vivos por residência da mãe, por ano. Após tabulação realizar cálculo da proporção para cada faixa de número de consultas.	Sinasc	Viés: numerador por ocorrência e denominador por residência
5	Razão do nº de atendimentos (individual e em grupo) realizados por psiquiatra na AB/ população	$$\frac{\mathrm{Atendimento}\;\mathrm{com}\;\mathrm{psiquiatra}\;(\mathrm{individual}\;e\;\mathrm{em}\;\mathrm{grupo})\;\mathrm{na}\;\mathrm{AB}\;\mathrm{por}\;\mathrm{local}\;\mathrm{de}\;\mathrm{atendimento}\;\mathrm{por}\;\mathrm{ano}}{\mathrm{População}\;\mathrm{total}\;(\mathrm{censo}\;e\;\mathrm{estimativas})\;x\;10.000}$$	SIA-SUS ou equivalente	Viés: numerador por ocorrência e denominador por residência
6	Razão do nº de atendimentos (individual e em grupo) realizados por psicólogo na AB/ população	$$\frac{\mathrm{Atendimento}\;\mathrm{com}\;\mathrm{psicólogo}\;(\mathrm{individual}\;e\;\mathrm{em}\;\mathrm{grupo})\;\mathrm{na}\;\mathrm{AB}\;\mathrm{por}\;\mathrm{local}\;\mathrm{de}\;\mathrm{atendimento}\;\mathrm{por}\;\mathrm{ano}}{\mathrm{População}\;\mathrm{total}\;(\mathrm{censo}\;e\;\mathrm{estimativas})\;x\;10.000}$$	SIA-SUS ou equivalente	Viés: numerador por ocorrência e denominador por residência
7	Razão de consulta especializada com cardiologista/ população	$$\frac{\mathrm{Número}\;\mathrm{de}\;\mathrm{consultas}\;\mathrm{com}\;\mathrm{médicos}\;\mathrm{cardiologista}\;+\;\mathrm{cardiologista}\;+\;\mathrm{cardiologista},\;\mathrm{médico}\;\mathrm{do}\;\mathrm{coração}\;\mathrm{realizadas}\;\mathrm{por}\;\mathrm{ano}}{\mathrm{População}\;\mathrm{total}\;\mathrm{por}\;\mathrm{ano}\;x\;1.000}$$	SIA-SUS	Viés: numerador por ocorrência e denominador por residência
8	Razão de ecocardiograma por 1.000 habitantes/ ano	$$\frac{\mathrm{Número}\;\mathrm{de}\;\mathrm{ecocardiografias}\;\mathrm{de}\;\mathrm{estresse}\;+\;\mathrm{ecocardiografias}\;\mathrm{transesofágica}\;+\;\mathrm{ecocardiografias}\;\mathrm{transtorácica}\;\mathrm{realizadas}\;\mathrm{por}\;\mathrm{ano}}{\mathrm{População}\;\mathrm{total}\;\mathrm{por}\;\mathrm{ano}\;x\;1.000}$$	SIA-SUS ou equivalente	Viés: numerador por ocorrência e denominador por residência
9	Razão de internação por hipertensão arterial por 10.000 habitantes	$$\frac{\mathrm{Número}\;\mathrm{de}\;\mathrm{internações}\;\mathrm{com}\;\mathrm{CID}\;\mathrm{principal}\;\mathrm{igual}\;a\;I10\;\mathrm{ou}\;I11}{\mathrm{População}\;\mathrm{total}\;\mathrm{por}\;\mathrm{ano}\;x\;10.000}$$	AIH-SIH	
10	Razão de mamografias realizadas na população de 50 a 69 anos ^a^	$$\frac{\mathrm{Número}\;\mathrm{de}\;\mathrm{mamografia}\;+\;\mathrm{marcação}\;\mathrm{pré}-\mathrm{cirúrgica}\;\mathrm{de}\;\mathrm{lesão}\;\mathrm{não}\;\mathrm{palpável}\;\mathrm{de}\;\mathrm{mama}\;\mathrm{associada}\;a\;\mathrm{mamografia}\;+\;\mathrm{mamografia}\;\mathrm{bilateral}\;\mathrm{para}\;\mathrm{rastreamento}\;\mathrm{em}\;\mathrm{mulheres}\;30\;a\;79\;\mathrm{anos}\;\mathrm{realizadas}\;\mathrm{por}\;\mathrm{ano}}{\mathrm{Número}\;\mathrm{de}\;\mathrm{mulheres}\;30\;a\;79\;\mathrm{anos}\;\mathrm{por}\;\mathrm{ano}}\;\times \;2$$	SIA-PA e IBGE censo e estimativas	
11	Proporção de mamografia Cat4 ou mais no total de mamografias realizadas	$$\frac{\mathrm{Soma}\;\mathrm{da}\;\mathrm{quantidade}\;\mathrm{anual}\;\mathrm{de}\;\mathrm{mamografias}\;\mathrm{cat}\;\mathrm{quatro}\;a\;\mathrm{seis}\;\mathrm{para}\;\mathrm{mulheres}\;\mathrm{na}\;\mathrm{faixa}\;\mathrm{etária}\;\mathrm{de}\;50\;\mathrm{anos}\;\mathrm{ou}\;\mathrm{mais}}{\mathrm{Número}\;\mathrm{total}\;\mathrm{das}\;\mathrm{mamografias}\;\mathrm{cat}\;0\;a\;6\;\mathrm{por}\;\mathrm{ano}\;\mathrm{para}\;\mathrm{mulheres}\;\mathrm{na}\;\mathrm{faixa}\;\mathrm{etária}\;\mathrm{de}\;50\;\mathrm{anos}\;\mathrm{ou}\;\mathrm{mais}\;x\;100}\;$$	Sismama/Siscan	
12	Razão de internação por câncer de mama em maiores de 50 anos e + /população feminina na faixa etária de 50 anos e mais	$$\frac{\mathrm{Número}\;\mathrm{de}\;\mathrm{internações}\;\mathrm{na}\;\mathrm{faixa}\;\mathrm{etária}\;\mathrm{de}\;50\;\mathrm{anos}\;\mathrm{ou}\;\mathrm{mais}\;\mathrm{com}\;\mathrm{CID}\;10\;\mathrm{neoplasia}\;\mathrm{maligna}\;\mathrm{da}\;\mathrm{mama}\;\mathrm{anual}}{\mathrm{Número}\;\mathrm{de}\;\mathrm{mulheres}\;\mathrm{de}\;50\;\mathrm{anos}\;\mathrm{ou}\;\mathrm{mais}\;x\;10.000}\;$$	IBGE e SIH	
13	Razão de consulta médica especializada com obstetra/ nascidos vivos	$$\frac{Nº\;\mathrm{de}\;\mathrm{consultas}\;\mathrm{médico}\;\mathrm{ginecologista}\;e\;\mathrm{obstetra}\;+\;\mathrm{médico}\;\mathrm{ginecologista}\;e\;\mathrm{obstetra}\;\mathrm{cirurgião}\;\mathrm{ginecológico}\;+\;\mathrm{médico}\;\mathrm{ginecologista}/\;\mathrm{obstetra}\;+\;\mathrm{médico}\;\mathrm{obstetra}\;\mathrm{em}\;\mathrm{consulta}\;\mathrm{médica}\;\mathrm{em}\;\mathrm{atenção}\;\mathrm{especializada}}{\mathrm{Número}\;\mathrm{total}\;\mathrm{de}\;\mathrm{nascidos}\;\mathrm{vivos}\;\mathrm{por}\;\mathrm{ano}}\;$$	Siasus ou equivalente e Sinasc	Viéses: 1) fator de confundimento: somadas consultas especializadas em ginecologia e obstetrícia 2) numerador por ocorrência e denominador por residência
14	Razão de US obstétrico morfológico/ nascidos vivos	$$\frac{\mathrm{Número}\;\mathrm{de}\;\mathrm{ultrassonogragia}\;\mathrm{doppler}\;\mathrm{de}\;\mathrm{fluxo}\;\mathrm{obstétrico}\;+\;\mathrm{ultrassonografia}\;\mathrm{obstétrica}\;+\;\mathrm{ultrassonografia}\;\mathrm{obstétrica}\;\mathrm{com}\;\mathrm{doppler}\;\mathrm{colorido}\;e\;\mathrm{pulsado}\;\mathrm{realizadas}\;\mathrm{por}\;\mathrm{ano}}{\mathrm{Número}\;\mathrm{total}\;\mathrm{de}\;\mathrm{nascidos}\;\mathrm{vivos}\;\mathrm{por}\;\mathrm{ano}}\;$$	Siasus ou equivalente e Sinasc	Viés: numerador por ocorrência e denominador por residência
15	Razão de Nº de partos em gestação de risco/nascidos vivos	$$\frac{\mathrm{Número}\;\mathrm{de}\;\mathrm{partos}\;\mathrm{normais}\;\mathrm{em}\;\mathrm{gestação}\;\mathrm{de}\;\mathrm{alto}\;\mathrm{risco}\;e\;\mathrm{de}\;\mathrm{parto}\;\mathrm{cesariano}\;\mathrm{em}\;\mathrm{gestação}\;\mathrm{alto}\;\mathrm{risco}\;\mathrm{por}\;\mathrm{ano}}{\mathrm{Número}\;\mathrm{total}\;\mathrm{de}\;\mathrm{nascidos}\;\mathrm{vivos}\;\mathrm{por}\;\mathrm{ano}\;x\;100}\;$$	AIH-SIH e Sinasc	
16	Proporção de Apgar maior ou igual a oito no quinto minuto/ nascidos vivos	$$\frac{\mathrm{Número}\;\mathrm{de}\;\mathrm{nascidos}\;\mathrm{vivos}\;\mathrm{por}\;\mathrm{residência}\;\mathrm{da}\;\mathrm{mãe},\;\mathrm{após}\;5º\;\mathrm{minuto}\;\mathrm{com}\;\mathrm{faixa}\;\mathrm{de}\;\mathrm{Apgar}\;8,\;9\;e\;10\;\mathrm{por}\;\mathrm{ano}}{\mathrm{Número}\;\mathrm{total}\;\mathrm{de}\;\mathrm{nascidos}\;\mathrm{vivos}\;\mathrm{por}\;\mathrm{residência}\;\mathrm{da}\;\mathrm{mãe},\;\mathrm{após}\;5º\;\mathrm{minuto}\;\mathrm{por}\;\mathrm{ano}}\;$$	Fonte: Sinasc	
17	Razão de número médio mensal de cadastros em Caps selecionados por 10.000 habitantes (20 anos e mais) por ano	$$\frac{\mathrm{Número}\;\mathrm{anual}\;\mathrm{de}\;\mathrm{pacientes}\;\mathrm{em}\;\mathrm{cada}\;\mathrm{Caps}\;\mathrm{selecionado}\;(\mathrm{exceto}\;\mathrm{Caps}\;\mathrm{AD}\;e\;\mathrm{Capsi})/12\;\mathrm{meses}}{\mathrm{População}\;\mathrm{estimada}\;\mathrm{no}\;\mathrm{ano}\;\mathrm{com}\;\mathrm{idade}\;20\;\mathrm{anos}\;x\;10.000}\;$$	SIA-RAAS	
18	Cobertura anual de Caps adulto pela população adulta	$$\frac{\mathrm{Número}\;\mathrm{anual}\;\mathrm{de}\;\mathrm{pacientes}\;\mathrm{em}\;\mathrm{cada}\;\mathrm{Caps}\;\mathrm{selecionado}\;(\mathrm{exceto}\;\mathrm{Caps}\;\mathrm{AD}\;e\;\mathrm{Capsi})}{\mathrm{População}\;\mathrm{estimada}\;\mathrm{no}\;\mathrm{ano}\;\mathrm{com}\;\mathrm{idade}\;20\;\mathrm{anos}\;x\;10.000}\;$$	SIA-RAAS	
19	Procedimento selecionado ACOLHIMENTO DIURNO DE PACIENTE EM CENTRO DE ATENÇÃO PSICOSSOCIAL por 10.000 habitantes/ano	$$\frac{\mathrm{Número}\;\mathrm{anual}\;\mathrm{de}\;\mathrm{acolhimento}\;\mathrm{diurno}\;\mathrm{de}\;\mathrm{pacientes}\;\mathrm{em}\;\mathrm{cada}\;\mathrm{Caps}\;\mathrm{selecionado}\;(\mathrm{exceto}\;\mathrm{Caps}\;\mathrm{AD}\;e\;\mathrm{Capsi})}{\mathrm{População}\;\mathrm{estimada}\;\mathrm{no}\;\mathrm{ano}\;\mathrm{com}\;\mathrm{idade}\;20\;\mathrm{anos}\;x\;10.000}\;$$	SIA-RAAS	Viés: numerador por ocorrência e denominador por residência
20	ATENDIMENTO EM GRUPO DE PACIENTE EM CENTRO DE ATENÇÃO PSICOSSOCIAL por 10.000 habitantes/ano em Caps	$$\frac{\mathrm{Número}\;\mathrm{anual}\;\mathrm{de}\;\mathrm{atendimento}\;\mathrm{de}\;\mathrm{grupo}\;\mathrm{de}\;\mathrm{pacientes}\;\mathrm{em}\;\mathrm{cada}\;\mathrm{Caps}\;\mathrm{selecionado}\;(\mathrm{exceto}\;\mathrm{Caps}\;\mathrm{AD}\;e\;\mathrm{Capsi})}{\mathrm{População}\;\mathrm{estimada}\;\mathrm{no}\;\mathrm{ano}\;\mathrm{com}\;\mathrm{idade}\;20\;\mathrm{anos}\;x\;10.000}\;$$	SIA-RAAS	Viés: numerador por ocorrência e denominador por residência
21	Procedimento selecionado ATENDIMENTO INDIVIDUAL DE PACIENTE EM CENTRO DE ATENÇÃO PSICOSSOCIAL por 10.000 habitantes/ano em Caps	$$\frac{\mathrm{Número}\;\mathrm{anual}\;\mathrm{de}\;\mathrm{atendimento}\;\mathrm{individual}\;\mathrm{de}\;\mathrm{pacientes}\;\mathrm{em}\;\mathrm{cada}\;\mathrm{Caps}\;\mathrm{selecionado}\;(\mathrm{exceto}\;\mathrm{Caps}\;\mathrm{AD}\;e\;\mathrm{Capsi})}{\mathrm{População}\;\mathrm{estimada}\;\mathrm{no}\;\mathrm{ano}\;\mathrm{com}\;\mathrm{idade}\;20\;\mathrm{anos}\;x\;10.000}\;$$	SIA-RAAS	Viés: numerador por ocorrência e denominador por residência
22	Proporção de atendidos que vieram da atenção básica	$$\frac{\mathrm{Número}\;\mathrm{médio}\;\mathrm{anual}\;\mathrm{de}\;\mathrm{pacientes}/\mathrm{mês}\;\mathrm{com}\;\mathrm{origem}\;\mathrm{na}\;\mathrm{AB}}{\mathrm{Número}\;\mathrm{médio}\;\mathrm{anual}\;\mathrm{de}\;\mathrm{pacientes}/\mathrm{mês}\;\mathrm{com}\;\mathrm{todas}\;\mathrm{as}\;\mathrm{origens}}\;$$	SIA-RAAS	
23	Razão de internações psiquiátricas SUS/ população (número de faturas mensais de AIH por 1.000 habitantes/ano)	$$\frac{\mathrm{Número}\;\mathrm{de}\;\mathrm{internações}\;\mathrm{psiquiátricas}\;e\;\mathrm{de}\;\mathrm{psiquiatria}\;\mathrm{em}\;\mathrm{hospital}\;\mathrm{geral}\;\mathrm{anual}}{\mathrm{População}\;\mathrm{total}\;\mathrm{estimada}\;\mathrm{no}\;\mathrm{ano}\;x\;1.000}\;$$	AIH-SIH e CNES	
24	Razão de internações SUS em hospital geral/ internações SUS em hospital psiquiátrico ^b^	$$\frac{\mathrm{Internações}\;\mathrm{em}\;\mathrm{hospital}\;\mathrm{geral}}{\mathrm{Internações}\;\mathrm{em}\;\mathrm{hospital}\;\mathrm{psiquiátrico}}\;$$	AIH-SIH e CNES	

^a^ Valor do resultado × 2, pois o parâmetro é um exame a cada dois anos.

^b^ Uso do Indicador 23.

AB: atenção básica; AIH: autorização de internação hospitalar; CAPS: centro de atenção psicossocial; CAPS AD: CAPS álcool e outras drogas; CAPSi: CAPS infanto-juvenil; CAT4: categoria 4; CID: Classificação Internacional de Doenças; CNES: Cadastro Nacional de Estabelecimentos de Saúde; ESF: Estratégia Saúde da Família; IBGE: Instituto Brasileiro de Geografia e Estatística; RAAS: Registro das Ações Ambulatoriais de Saúde; SIA-SUS: Sistema de Informações Ambulatoriais do SUS; SIH: Sistema de Informações hospitalares; SINASC: Sistema de Informações sobre Nascidos Vivos; SISCAN: Sistema de Informação do Câncer; SISMAMA: Sistema de Informação do câncer e mama; SUS: Sistema Único de Saúde; US: ultrassonografia.

Nessa abordagem inicial de dados secundários, foi possível identificar diferenças nas redes de serviços existentes em cada município para cada agravo específico. A análise crítica das relações existentes entre os distintos procedimentos esperados na linha de cuidado de cada agravo permitiu diferenciar o tipo de oferta e o papel da regulação na atenção aos agravos em cada município.

## O Complemento Qualitativo-Descritivo

Um estudo descritivo-qualitativo foi realizado para cada cidade, buscando uma descrição densa da rede de serviços de cada agravo, singularidades de seu funcionamento e eventos históricos mais relevantes. Para tanto, construiu-se um esquema-padrão, validado coletivamente pela equipe de pesquisa. Cada um dos quesitos foi preenchido por um grupo de pesquisadores de cada localidade, com base nas fontes disponíveis e recorrendo a informantes-chave quando necessário.

As questões elencadas incluíram informações sobre o funcionamento da atenção básica, disponibilidade e composição dos Núcleos de Apoio à Saúde da Família (NASF) e equipes de apoio em saúde mental, modo de marcação de consultas especializadas, uso de referência/contrarreferência, forma de organização das redes temáticas de cada agravo, disponibilidade de serviços universitários integrados ou não ao SUS, e forma de regulação de leitos para as clínicas relevantes a cada agravo. O conjunto das informações contidas no esquema para cada agravo e cidade encontra-se no Quadro 2 .

**Quadro 2:** Informações do esquema qualitativo

Rede pública e conveniada
Funcionamento da atenção básica (ESF, misto, programático, pronto-atendimento, forma de encaixe de intercorrências, uso de avaliação de risco com ou sem acolhimento)
Rede temática (existe para esse agravo? como ocorre o acesso inicial e o trânsito aos demais pontos de atenção?)
Há apoio matricial/NASF de quais especialidades?
Há serviços universitários (hospitalares, pronto-atendimento)? Ocorre alguma regulação de vagas pela prefeitura?
Descrição da central de marcação de consultas
Descrição da central de regulação de vagas
Outras características singulares da rede local e sua história que mereçam ser detalhadas

ESF: Estratégia Saúde da Família; NASF: Núcleo de Apoio à Saúde da Família.

Essas informações qualitativas permitiram aprofundar a compreensão de cada contexto local e subsidiaram uma análise complementar das magnitudes e tendências evidenciadas pelos indicadores derivados dos sistemas de informação, constituindo uma primeira rodada de triangulação.

## Inquérito de Serviços: Desenho, Amostragem, Plano de Análise

Para o estudo transversal com usuários dos serviços de referência, foram escolhidas as seguintes modalidades de serviços:

- Câncer de mama: centros de alta complexidade em oncologia.
- Saúde mental: Centros de Atenção Psicossocial (CAPS);
- Hipertensão arterial: ambulatórios de cardiologia.
- Gravidez de risco: maternidades, ambulatórios de obstetrícia.

A opção pelos serviços especializados deu-se por assegurar maior representatividade da rede de saúde de cada cidade: torna-se possível abordar todos os serviços especializados, uma vez que são poucos, mas recebem usuários da atenção básica do município inteiro. Dessa forma, desenvolvemos quatro questionários abordando aspectos da APS e atenção especializada referentes a cada agravo, aplicados em usuários em acompanhamento na atenção especializada.

Os questionários, particularizados para cada agravo, contendo entre 49 e 66 perguntas, visaram identificar e mensurar eventos referentes à trajetória dos usuários nos serviços de atenção primária e especializada. Tais eventos eram fundamentalmente boas práticas protocolizadas ou fundamentadas na literatura científica da área, tais como intervenções e condutas, assim como tempo de espera, frequência e local (APS ou atenção especializada) de sua realização.

Os quatro questionários partilham seis eixos que norteiam suas perguntas: 1) informações sociodemográficas; 2) características da atenção especializada; 3) características da atenção primária; 4) medicação, exames complementares e orientações, principalmente no trajeto atenção primária-especializada; 5) utilização de urgência e emergência; e 6) utilização de convênios e serviços privados. A versão integral pode ser acessada no website da pesquisa ^a^ .

^a^ Universidade Estadual de Campinas, Faculdade de Ciências Médicas, Núcleo de Tecnologia da Informação. AcesSUS: metodologia. Campinas, SP: FCM Unicamp; s.d. [citado 18 jul 2020]. Disponível em: https://www.fcm.unicamp.br/acessus/metodologia

### Amostragem

Foram utilizadas abordagens diferentes de amostragem de acordo com particularidades de cada município e agravo. Foram entrevistados 7.053 usuários, divididos nas quatro condições traçadoras. O quadro 3 resume as estratégias utilizadas. Abordagem detalhada dos métodos de aleatorização pode ser encontrada no website da pesquisa ^a^ .

**Quadro 3:** Estratégias de amostragem para cada agravo e município.

Município	Agravo	Tipo de amostragem	Serviços no município	Serviços participantes pesquisa	Total de inquéritos
Campinas	Hipertensão arterial	Amostra simples	4	4	485
Fortaleza	Hipertensão arterial	Amostra simples	5	5	417
Porto Alegre	Hipertensão arterial	Amostra simples	9	4	408
São Paulo	Hipertensão arterial	Amostragem por conglomerados em dois estágios (serviços e usuários)	52	30	760
Campinas	Gestação de risco	Amostra simples	3	3	500
Fortaleza	Gestação de risco	Amostra simples	5	5	401
Porto Alegre	Gestação de risco	Amostra simples	5	5	391
São Paulo	Gestação de risco	Amostra simples	15	15	689
Campinas	Câncer de mama	Amostra simples	3	2	318
Fortaleza	Câncer de mama	Amostra simples	5	5	334
Porto Alegre	Câncer de mama	Amostra simples	5	5	355
São Paulo	Câncer de mama	Amostra simples	4	1	353
Campinas	Saúde mental	Censo	6	6	393
Fortaleza	Saúde mental	Censo	6	6	601
Porto Alegre	Saúde mental	Censo	4	4	351
São Paulo	Saúde mental	Amostra simples	34	24	297

### Plano de Análise

A primeira análise dos dados coletados nos questionários visou identificar diferenças significativas entre os eventos captados. Estas diferenças foram analisadas em relação às características sociodemográficas para permitir uma análise mais adequada das relações significativas. Complementando estas análises bivariadas, foram realizadas análises multivariadas, visando controlar variáveis intervenientes ou correlacionadas. Buscamos analisar essas associações contemplando algumas dimensões: acesso, vínculo e cuidado.

Nesse momento, para cada agravo algumas questões puderam ser esclarecidas por um novo cruzamento com os indicadores obtidos a partir dos dados secundários (segunda triangulação).

## O Complemento Qualitativo-Analítico

Após essa sequência de procedimentos, focos de desenho qualitativo foram explorados para aumentar a capacidade de análise quando os dados das etapas anteriores apresentassem necessidade de aprofundamento.

Lançou-se mão de entrevistas em profundidade para atores-chave, registro dos itinerários terapêuticos de usuários, narrativas de história de vida e busca ativa de perdas dos serviços especializados, de acordo com cada caso. Esses focos de estudos qualitativos permitiram aprofundar questões, interpretar correlações incompreensíveis e aumentar a capacidade analítica do desenho.

## DISCUSSÃO

Argumentamos que o desenho multifacetado e de métodos mistos possibilitou interessante aproximação à complexidade real do sistema de saúde brasileiro, propiciando uma análise explicativa por meio da combinação sequencial e concomitante de abordagens complementares.

O estudo valeu-se de dados quantitativos e ampla variedade de entrevistados. As fases sequenciais de análise permitiram um engajamento de longo prazo dos pesquisadores e repetidas checagens de dados e reinterpretações. As informações qualitativas enriqueceram a interpretação das análises estatísticas, qualificando inclusive alguns achados pouco claros do inquérito nos serviços. Essa triangulação de métodos permitiu ampliar e aprofundar a investigação das temáticas em pauta, estratégia ainda incomum em estudos de políticas e sistemas de saúde, apesar de recomendada na literatura da área ^4 , 5 , 8^ . Destacamos a seguir alguns pontos específicos que evidenciam as vantagens dessa triangulação.

A sequência de etapas no plano de análise propiciou maior sensibilidade em relação às desigualdades regionais e às especificidades de cada uma das cidades estudadas. A consideração inicial de cada município como um estudo de caso autônomo permitiu compreender a história e o contexto local, desvelando alterações súbitas em indicadores importantes em determinadas cidades, em anos específicos, assim como prioridades históricas na construção de determinados modelos de atenção.

Em relação aos indicadores sensíveis às prioridades das gestões locais, identificou-se, por exemplo, mudanças na cobertura de APS, na proporção entre APS tradicional e ESF, e na provisão de equipes NASF. Verificou-se ainda estabilidade ou oscilações em procedimentos específicos de cada agravo traçador, na APS e/ou na atenção especializada.

As entrevistas com informantes-chave, que subsidiaram a máscara qualitativa-descritiva, permitiram aproximar as vozes de outros atores, engajados nas instâncias de gestão e administração dos serviços, procedimento cuja importância já foi salientada por outros estudos ^3^ .

Esse conjunto de informações qualificou as percepções acerca dos contextos específicos, a partir dos quais ocorreu o acesso à atenção especializada por meio da APS de cada município, para cada agravo. Em síntese, as séries temporais de dados secundários e indicadores contribuíram para contextualizar historicamente os resultados do estudo transversal e componente qualitativo. As entrevistas qualitativas, por sua vez, complementaram a análise estatística do estudo transversal em uma nova rodada da espiral de análise dos dados.

Consideramos que, mesmo com um componente quantitativo predominante em nossa pesquisa, as informações produzidas encontram-se mais próximas do modelo “qualitativo” de generalização teórica ^7^ . A partir disso, a comparação entre os achados de cada agravo, em cada município, permitiu encontrar regularidades que ocorrem de forma transversal nos diferentes focos de análise (agravo e município), mas também efeitos isolados, de *loci* específicos.

A necessidade de contextualizar nossos achados criou uma tarefa diferente da dos estudos epidemiológicos tradicionais – cuja preocupação maior é isolar variáveis que têm efeitos significativos sobre outras variáveis –, uma vez que não seria possível isolar nossas variáveis de interesse fora do contexto de cada rede e cidade, uma vez que estávamos interessados em entender tais funcionamentos singulares.

Tradicionalmente, a preocupação com o contexto dos achados se traduz como interpretações dos significados culturalmente atribuídos a determinado fenômeno. Ainda que tal concepção tenha estado presente em nosso componente qualitativo, destacamos que a caracterização do contexto de cada município passou também pelo contraste entre a estatística descritiva e analítica realizada nos indicadores utilizados na pesquisa, assim como nos resultados dos inquéritos em serviço.

Segundo a literatura, a dificuldade de reduzir fatores contextuais relevantes a um conjunto de medidas quantificáveis tem gerado descrições densas, comuns em referenciais antropológicos ^8^ . Contudo, nossa triangulação permitiu abordar o contexto por meio de duas vertentes distintas, incluindo na análise as experiências particulares das pessoas envolvidas, produzindo descrições densas, em conjunção com elementos objetivos mensurados quantitativamente, criando assim diferentes “camadas” de contexto.

Destacamos, por exemplo, associações entre variáveis que ocorriam apenas em algumas das cidades investigadas, o que permitiu identificar as diferentes performances do modo de funcionamento de cada rede local a serem aprofundadas no componente qualitativo.

A capacidade de generalização plena de evidências científicas tem sido questionada. Revisões sistemáticas de ensaios clínicos randomizados têm trazido informações conflitantes sobre a efetividade de intervenções, destacando que determinadas ações, que demonstram efeitos em um cenário específico de pesquisa ou de implementação, não necessariamente apresentam os mesmos resultados em um cenário diferente ^5 , 31^ .

Ainda que a compreensão da subjetividade seja um dos elementos centrais da pesquisa qualitativa, na pesquisa clínica de epistemologia quantitativa o uso dos *patient-reported outcomes* também tem sido advogado, especialmente quando os resultados a serem mensurados não permitem observação direta ^32 , 33^ . Reconhece-se a importância de uma série de fenômenos complexos, que só podem ser acessados a partir do relato do próprio paciente, seja por meio de entrevistas, questionários ou escalas para captar de forma comparativa a percepção do objeto da pesquisa pelos diferentes grupos de interesse.

Em concordância com essas premissas, desenvolvemos um inquérito com os usuários dos serviços especializados, incorporando as vantagens dessa abordagem, porém reconhecendo suas limitações. A principal vantagem foi permitir a possibilidade de análises intrasserviço, entre serviços e entre municípios, para cada agravo investigado, respeitando-se os diferentes níveis de significância estatística em cada análise comparativa.

Em relação às limitações do inquérito transversal, observa-se na literatura tanto superestimação como subestimação da utilização de serviços em questionários autorreferidos ^32^ . Eventos rememorados após 12 meses e eventos recorrentes tendem a ser mais frequentemente subestimados; eventos raros e marcantes, como hospitalizações, apresentam pouco viés de memória. Também se observa variação entre as informações dos diagnósticos em saúde mental autorrelatados em relação aos registros em prontuário médico ^34^ , viés que também pode ter influenciado nosso estudo.

Apesar dessas limitações, consideramos que o inquérito com usuários de serviços é capaz de produzir informações insubstituíveis, auxiliando a corrigir fragilidades decorrentes do sub-registro nos sistemas de informações, assim como abordando minúcias não catalogadas por esses sistemas.

## CONSIDERAÇÕES FINAIS

O modelo analítico multifacetado apresentado permitiu compreender o funcionamento de um sistema de saúde público de caráter universal em um país de renda média, atentando para as singularidades, heterogeneidades e desigualdades que caracterizam a implantação desse sistema no Brasil.

As principais características deste construto foram a utilização de condições traçadoras para direcionar o plano de análise, tendo como diretrizes as linhas de cuidado específicas, assim como a utilização dos dados secundários disponíveis, permitindo a aproximação a séries temporais e a formulação preliminar de hipóteses de relações.

Destaca-se também a abordagem de métodos mistos, que permitiu incorporar grande número e ampla gama de entrevistados em diferentes níveis do sistema, com envolvimento recorrente junto às partes interessadas, permitindo verificar dados e reinterpretar as análises quando necessário. A abordagem qualitativa propiciou identificar variáveis locais que favoreciam ou dificultavam o cuidado longitudinal e a regulação da rede de serviços pela APS nos agravos de saúde estudados.

O âmbito geográfico abrangente e a diversidade de locais estudados permitiram analisar a performance do SUS em relação aos padrões de boas práticas para os agravos estudados, ainda que sem reivindicar representatividade e generalização a todo o Brasil.

Por fim, o uso de um quadro multifacetado de técnicas de pesquisa permitiu compreender várias dimensões do acesso aos cuidados, com sensibilidade para a detecção de formatos excessivamente centrados nos especialistas, contextualizando as informações disponíveis nos sistemas de informação.

Esperamos que o presente desenho contribua com outras pesquisas que buscam dar conta da variabilidade da implantação do SUS no território nacional. O desenho de abordagem quanti-qualitativa concomitante e sequencial, utilizando condições traçadoras, aumentou a capacidade analítica da abordagem, inseriu variados informantes e permitiu análises temporais. Ao contemplar heterogeneidades e particularidades, tal desenho também propiciou uma compreensão mais ampliada do funcionamento das redes locais nos agravos estudados. Consideramos que nesses pontos residem as principais contribuições da estratégia de pesquisa apresentada à análise de sistemas de saúde.
